# Nocturnal Hypoxemia Rather Than Obstructive Sleep Apnea Is Associated With Decreased Red Blood Cell Deformability and Enhanced Hemolysis in Patients With Sickle Cell Disease

**DOI:** 10.3389/fphys.2021.743399

**Published:** 2021-09-24

**Authors:** Emeric Stauffer, Solène Poutrel, Giovanna Cannas, Alexandra Gauthier, Romain Fort, Yves Bertrand, Céline Renoux, Philippe Joly, Camille Boisson, Arnaud Hot, Laure Peter-Derex, Vincent Pialoux, Thierry PetitJean, Philippe Connes

**Affiliations:** ^1^Laboratoire Interuniversitaire de Biologie de la Motricité (LIBM) EA7424, Team ≪ Vascular Biology and Red Blood Cell ≫, University Claude Bernard Lyon 1, Lyon, France; ^2^Laboratoire d'Excellence du Globule Rouge (Labex GR-Ex), PRES Sorbonne, Paris, France; ^3^Centre de Médecine du Sommeil et des Maladies Respiratoires, Hospices Civils de Lyon, Hôpital Croix Rousse, Lyon, France; ^4^Service d'Explorations Fonctionnelles Respiratoires-Médecine du sport et de l'activité physique, Hospices Civils de Lyon, Lyon, France; ^5^Service de Médecine Interne, Hôpital Edouard Herriot, Hospices Civils de Lyon, Lyon, France; ^6^Institut d'Hématologique et d'Oncologique Pédiatrique, Hospices Civils de Lyon, Lyon, France; ^7^Service de Réanimation Médicale, Hôpital Lyon Sud, Hospices Civils de Lyon, Lyon, France; ^8^Laboratoire de Biochimie et de Biologie Moléculaire, UF de Biochimie des Pathologies Erythrocytaires, Centre de Biologie et de Pathologie Est, Hospices Civils de Lyon, Lyon, France; ^9^Centre de recherche en Neurosciences de Lyon, CNRS UMR 5292, INSERM U 1028, Universite Claude Bernard Lyon 1, Lyon, France

**Keywords:** sickle cell disease, nocturnal hypoxemia, hemolysis, obstructive sleep apnea, red blood cell deformability

## Abstract

**Background:** Although obstructive sleep apnea (OSA) could act as a modulator of clinical severity in sickle cell disease (SCD), few studies focused on the associations between the two diseases.

**Research Question:** The aims of this study were: (1) to explore the associations between OSA, nocturnal oxyhemoglobin saturation (SpO2) and the history of several acute/chronic complications, (2) to investigate the impact of OSA and nocturnal SpO2 on several biomarkers (hematological, blood rheological, and coagulation) in patients with SCD.

**Study Design and Methods:** Forty-three homozygous SCD patients underwent a complete polysomnography recording followed by blood sampling.

**Results:** The proportion of patients suffering from nocturnal hypoxemia did not differ between those with and those without OSA. No association between OSA and clinical severity was found. Nocturnal hypoxemia was associated with a higher proportion of patients with hemolytic complications (glomerulopathy, leg ulcer, priapism, or pulmonary hypertension). In addition, nocturnal hypoxemia was accompanied by a decrease in RBC deformability, enhanced hemolysis and more severe anemia.

**Interpretation:** Nocturnal hypoxemia in SCD patients could be responsible for changes in RBC deformability resulting in enhanced hemolysis leading to the development of complications such as leg ulcers, priapism, pulmonary hypertension or glomerulopathy.

**Clinical Trial Registration:** www.ClinicalTrials.gov, identifier: NCT03753854.

## Introduction

Sickle cell disease (SCD) is the most frequent genetic disease in the world, and is recognized as a public health priority in France. SCD is an autosomal recessive disorder resulting from a single mutation in the β-globin gene, leading to the production of an abnormal hemoglobin, hemoglobin S (HbS). HbS in red blood cells (RBCs) from SCD patients accounts for ~80–90% of the total hemoglobin content. In contrast to normal hemoglobin, HbS may form aggregates under deoxygenation, which results in a mechanical distortion of RBCs into a crescent-like shape. Sickled RBCs are very fragile and rigid, which may explain why HbSS patients are characterized by chronic hemolytic anemia, and may experience frequent and repeated vaso-occlusive crises.

Sleep-disordered breathing (SDB), and more particularly obstructive sleep apnea (OSA), occurs at a high frequency in SCD population (Gileles-Hillel et al., 2015; Raghunathan et al., 2018). For instance, Katz et al. (2018) and Sharma et al. (2015) reported that 22% of SCD children and 40% of SCD adults had OSA, respectively. Although OSA could act as a modulator of clinical severity in SCD, studies focusing on the associations of the two diseases are scarce (Gileles-Hillel et al., 2015; Ruhl et al., 2019). OSA may compromise respiratory gas exchange during sleep and cause oxyhemoglobin desaturation. As a result, OSA has been suspected to trigger vaso-occlusive like events (Hargrave et al., 2003; Gileles-Hillel et al., 2015; Brennan et al., 2020). However, studies focusing on the association between OSA and the frequency of vaso-occlusive like events reported inconsistent results. Katz et al. (2018) found an association between the frequency of acute chest syndrome and OSA. In contrast, a recent study performed in SCD children failed to find an association between OSA or low nocturnal oxyhemoglobin saturation (SpO2) and the frequency of vaso-occlusive like events (Willen et al., 2018). Low nocturnal oxyhemoglobin saturation has been associated with biomarkers of hemolysis and endothelial activation in SCD (Setty et al., 2003; Rotz et al., 2016), and enhanced hemolysis plays a central role in the pathophysiology of SCD (Nader et al., 2021). However, the associations between complications related to chronic hemolysis and low oxyhemoglobin saturation have not been investigated.

The aim of the present study was to investigate the associations between OSA, nocturnal oxyhemoglobin saturation and the rates of several acute/chronic complications. In addition, we tested the impact of OSA and nocturnal oxyhemoglobin saturation on several biomarkers (hematological, blood rheological, and coagulation) known to be involved in the pathophysiology of SCD, to gain further insights into the role of SDB in SCD.

## Materials and Methods

### Population

Sixty patients with homozygous SCD (i.e., HbSS genotype) and routinely followed at the Sickle Center of the Hospices Civils de Lyon were eligible to participate to the study. The inclusions criteria were: age between 15 and 55, symptoms of OSA (excessive sleepiness, snoring, daytime fatigue, feeling of suffocation during sleep, morning headache, nocturia). Seventeen subjects were not interested in participating in the study. Forty-three SCD patients (22 men and 21 women; ages ranging from 15 to 52 years) were prospectively recruited for this study between 2018 and 2020. They were all at steady-state, i.e., without any transfusion or acute event requiring hospitalization in the 2 months preceding their visit. None of the patients included in this study had experienced overt stroke or abnormal transcranial doppler ultrasonography. All patients underwent a full night polysomnography recording (PSG) at the Sleep Medicine and Respiratory Diseases department of the Hospices Civils de Lyon. The rate of hospitalized vaso-occlusive crises (VOC) and acute chest syndrome (ACS) was retrospectively calculated over the 2-year period preceding the PSG. All patients were routinely followed at the Sickle Center and all clinical events requiring hospitalization are consigned in their clinical charts. Clinical charts were carefully reviewed to identify the presence of other complications at the time of the study such as priapism, glomerulopathy, pulmonary hypertension, leg ulcer, osteonecrosis, and retinopathy. Common definition was used to define each of this complication (Ballas, 2018). Clinical exam included anthropometric, heart rate (HR), diurnal SpO_2_, systolic (SBP) and diastolic (DBP) blood pressure measurements. The study was approved by the ethics committee (CPP Lyon OUEST V; 2017-A03352-51; ClinicalTrials number: NCT03753854) and performed according to the Declaration of Helsinki.

### Polysomnography Recording

The PSG was performed according to the American Academy of Sleep Medicine (AASM) (Kapur et al., 2017). Several signals were recorded: electroencephalogram, electrooculogram, chin and tibialis electromyogram, electrocardiogram, nasal airflow (nasal pressure and thermistor), pulse oximetry, microphone, and respiratory efforts (thoracic and abdominal). All the recordings were interpreted by the same sleep disorders specialist. Nocturnal hypoxemia was defined as more than 10% of total sleep time below a SpO2 of 90% (Deflandre et al., 2018). According to the AASM, apnea-hypopnea index (AHI) was defined by the sum of apnea (peak signal excursion drop by ≥90% for ≥10 s) and hypopnea (peak signal excursion drop by ≥30% for ≥10 s and ≥3% oxygen desaturation or event associated with arousal) per hour of sleep. Apnea (number of apnea per hour of sleep) and hypopnea index (number of hypopnea per hour of sleep) were also measured. OSA was defined as an AHI >5 per hour (Kapur et al., 2017). Radial arterial partial pressure in oxygen (PaO_2_) was measured in the morning, after PSG recording ended, when the patient awoke.

### Lung Function

All subjects underwent spirometry to determine their forced vital capacity (FVC), forced expiratory volume in 1 second (FEV_1_), FEV_1_/FVC and forced inspiratory flow at 25–75% of FVC (FEF25–75%). Lung volumes were also measured: total lung capacity (TLC), residual volume (RV), functional residual capacity (FRC), alveolar volume (AV), and inspiratory volume (IV). Finally, diffusing capacity for carbon monoxide (DLCO) was assessed. The values were corrected for hemoglobin concentration (Graham et al., 2017). The single breath method was used to determine AV and derive the carbon monoxide transfer coefficient (KCO), which represents an index of the efficiency of the alveolar transfer of carbon monoxide (Graham et al., 2017). Predicted values for FEV1, FVC, FEV1/FVC, TLC, RV, and DLCO were calculated on the basis of established algorithms taking into account gender, age, and height in the African population (Stanojevic et al., 2017).

### Biological Parameters

Blood samples were collected in the morning following the PSG when the patient awoke to measure several hematological [hemoglobin concentration (Hb); hematocrit (Hct); neutrophils, monocytes and platelets count; percentage of reticulocytes], biochemical [lactate dehydrogenase (LDH), total and free bilirubin] and coagulation (C protein, S protein and D-dimer) markers. Blood viscosity was measured at 22.5 and 45 s^−1^ using a cone plate viscometer (Brookfield DVII with CPE40 spindle, Ametek Brookfield, Middleborough, USA), according to the guidelines for hemorheological laboratory techniques (Baskurt et al., 2009). Red blood cell (RBC) deformability index was measured by ektacytometry using a Laser Optical Rotational Red Cell Analyzer (LORRCA, RR Mechatronics, Hoorn, The Netherlands) at 3 and 30 Pa. The technique has been described in details elsewhere (Baskurt et al., 2009; Renoux et al., 2016).

### Statistical Analysis

All data are expressed as mean ± SD. Statistical analyses were performed using IBM SPSS version 22. Qualitative analyses were performed by using a χ^2^ test. Unpaired student *t* tests were used to compare the different groups (with vs. without OSA; with vs. without nocturnal hypoxemia). A *p* value < 0.05 was considered statistically significant.

## Results

### Clinical Characteristics

Seven patients suffered from nocturnal hypoxemia (16%) and twenty-nine from OSA (67%) with 17 having mild OSA (AHI ≥ 5 but <15) and 12 exhibiting moderate (AHI ≥ 15 but <30; *n* = 6) to severe OSA (AHI ≥ 30; *n* = 6). The proportion of patients suffering from nocturnal hypoxemia did not differ between those with OSA (57.1%) and those without (42.9%; χ^2^ = 0.40, *p* = 0.665). No significant correlation was found between AHI and mean nocturnal SpO2 (*r* = −0.27; *p* = 0.076), minimum nocturnal SpO2 (*r* = −0.27; *p* = 0.076) or the percentage of time with a SpO2 lower than 90% (*r* = 0.21; *p* = 0.175). No significant correlation was observed between mean nocturnal SpO2 and HbF (*r* = 0.079; *p* = 0.616). Two patients (4.5%) had glomerulopathy, 1 had leg ulcer (2.3%), 5 had priapism (11.4%), 3 had pulmonary hypertension determined by echocardiography (6.8%), 13 had retinopathy (29.5%), 16 had osteonecrosis (36.4%), and 30 were under hydroxyurea (HU) medication (68.2%). Sixty-three percent of patients had experienced at least 1 VOC and 19% at least 1 ACS in the two preceding years.

#### Effects of Nocturnal Hypoxemia

Patients suffering from nocturnal hypoxemia exhibited lower diurnal Spo2 and lower PaO_2_ at the end of the PSG exam (Table 1). The proportion of patients under HU was not different between those with (71.4%) and those without (69.4%; χ^2^ = 0.01, *p* = 0.92) nocturnal hypoxemia. There was no significant difference between nocturnal hypoxemic and non-hypoxemic patients regarding age, weight, height, body mass index (BMI), heart rate and blood pressure (Table 1). Because some of the complications were rather rare, we grouped the different complications according to the “hemolytic vs. vaso-occlusive” phenotypes (Kato et al., 2007) with glomerulopathy, leg ulcer, priapism and pulmonary hypertension belonging to the “hemolytic complications” and VOC, ACS, osteonecrosis and retinopathy belonging to the “vaso-occlusive like complications.” The proportion of patients with a positive history of vaso-occlusive like complications did not differ between those with nocturnal hypoxemia and those without (χ^2^ = 0.057, *p* = 1; Table 1). In contrast, hemolytic complications were more frequent in the hypoxemic than in the non-hypoxemic group (χ^2^ = 6.625, *p* = 0.026). Fourteen patients had hydroxyurea treatment (30%). The proportion of patients treated with hydroxyurea did not differ between those with and those without hemolytic complications (χ^2^ = 1.973, *p* = 0.237). Lung function test showed no difference in spirometry, lung volume, and pulmonary diffusion capacity between the two groups.

**Table 1:** Clinical and lung function characteristics according to nocturnal hypoxemia.

	**<10% with SpO2 <90%**	**≥10% with SpO2 <90%**	***p***
	**(*n* = 36)**	**(*n* = 7)**	
Age (years)	28.9 ± 9	24.7 ± 10.4	ns
Weight (kg)	63.8 ± 8.7	61.7 ± 23.6	ns
Height (cm)	170.5 ± 8.9	169.6 ± 7.2	ns
BMI (kg/m^2^)	21.8 ± 2.9	21.5 ± 8.6	ns
Heart rate (bpm)	77.6 ± 12	84.6 ± 12.1	ns
SBP (mmHg)	118.7 ± 17.4	121.3 ± 9	ns
DBP (mmHg)	66.8 ± 10.6	64.9 ± 8.1	ns
Diurnal SpO2 (%)	97.7 ± 2.0	95.7 ± 2.0	**0.026**
Waking PaO2 (mmHg)	89.4 ± 18.1	74.6 ± 11	**0.022**
Mean sleep SpO2	94.8 ± 2.1	87.6 ± 2.8	** <0.001**
Hemolytic like complications (%)	14	57	**0.026**
Vaso occlusive like complication (%)	89	86	ns
Hydroxyurea treatment (%)	69	71	ns
FVC (L)	3.1 ± 0.8	2.9 ± 0.7	ns
FEV1 (L)	2.7 ± 0.6	2.4 ± 0.6	ns
FEV1/FVC	82.3 ± 7.7	83.9 ± 6.4	ns
TLC (L)	4.6 ± 1.3	4.1 ± 2	ns
RV (L)	1.5 ± 0.5	1.7 ± 0.5	ns
RV/TLC	32.2 ± 7.2	35.4 ± 5.3	ns
DLCO [mL/(min x mmHg)]	21.1 ± 6.3	18.1 ± 8.5	ns
KCO (mL/(min x mmHg x L))	5.1 ± 1.3	4.7 ± 2.5	ns
AV (L)	4 ± 0.9	3.8 ± 0.9	ns

*BMI, body mass index; SBP, systolic blood pressure; DBP, diastolic blood pressure; PaO_2_,arterial partial pressure of oxygen; FVC, forced vital capacity; FEV1, forced expiratory volume in one second; RV, Residual Volume; TLC, Total Lung Capacity; DLCO, diffusing capacity for carbon monoxide; KCO, DLCO/AV; AV, Alveolar Volume; ns, non significant. Bold p values mean Statistically significant*.

#### Effects of OSA

Hypopneas were much more common than apneas in patients suffering from OSA: mean hypopnea index was 21 ± 22.1 and apnea index was 1.3 ± 2.1. The hypopnea mean duration was 21.1 ± 4.1 s and the apnea mean duration was 14.7 ± 5.4 s.

The proportion of patients under HU was not different between those with (69.0%) and those without (71.4%; χ^2^ = 0.027, *p* = 0.87; Table 2) OSA. There was no significant difference between OSA and non-OSA patients regarding age, weight, height, BMI, pulse blood pressure, SpO_2_, and PaO_2_ (Table 2). Neither the proportion of patients with vaso-occlusive like complications (χ^2^ = 2.73, *p* = 0.156), nor the proportion of patients with hemolytic like complications differed between patients with or without OSA (χ^2^ = 0.554, *p* = 0.693). Lung function test showed no difference in spirometry, lung volume, and pulmonary diffusion capacity between the two groups.

**Table 2:** Clinical and lung function characteristics according to OSA.

	**Non-OSA (*n* = 14)**	**OSA (*n* = 29)**	***p***
Age (years)	27.3 ± 6.8	28.6 ± 10.3	ns
Weight (kg)	62.9 ± 9.2	63.7 ± 13.3	ns
Height (cm)	170.4 ± 9.5	170.3 ± 8	ns
BMI (kg/m^2^)	21.7 ± 3	21.8 ± 4.8	ns
Heart rate (bpm)	83.4 ± 10	76.6 ± 12.6	ns
SBP (mmHg)	119.1 ± 14.7	119.2 ± 17.1	ns
DBP (mmHg)	69.2 ± 7.9	65.2 ± 11	ns
Diurnal SpO2 (%)	97.3 ± 1.8	97.3 ± 2.3	ns
Waking PaO2 (mmHg)	84.7 ± 11.8	88.1 ± 20.3	ns
Mean sleep SpO2	93.4 ± 2.9	93.7 ± 3.7	ns
Hemolytic like complications (%)	14	24	ns
Vaso occlusive like complication (%)	100	83	ns
Hydroxyurea treatment (%)	71	68	ns
FVC (L)	3.1 ± 1	3 ± 0.7	ns
FEV1 (L)	2.6 ± 0.8	2.6 ± 0.6	ns
FEV1/FVC	80.8 ± 5.3	83.4 ± 8.2	ns
TLC (L)	4.8 ± 1	4.3 ± 1.5	ns
VR (L)	1.6 ± 0.5	1.5 ± 0.4	ns
VR/TLC	32.7 ± 9	32.6 ± 6.1	ns
DLCO [mL/(min x mmHg)]	19.5 ± 8.6	21.1 ± 5.8	ns
KCO [mL/(min x mmHg x L)]	5 ± 0.8	5.1 ± 1.8	ns
VA (L)	4 ± 1	3.9 ± 0.8	ns

*BMI, body mass index; SBP, systolic blood pressure; DBP, diastolic blood pressure; PaO_2_, arterial partial pressure of oxygen; FVC, forced vital capacity; FEV1, forced expiratory volume in one second; RV, Residual Volume; TLC, Total Lung Capacity; DLCO, diffusing capacity for carbon monoxide; KCO, DLCO/AV; AV, Alveolar Volume; ns, non significant*.

### Biological Parameters

#### Effects of Nocturnal Hypoxemia

Data are displayed in Table 3. Patients suffering from nocturnal hypoxemia exhibited lower Hb and Ht, lower RBC deformability at both 3 and 30 Pa, and lower blood viscosity at both 22.5 s^−1^ and 45 s^−1^ compared to the non-hypoxemic group. The hypoxemic group also had higher reticulocytes, neutrophils and monocytes count, free bilirubin, and LDH levels. Both RBC deformability at 3 and 30 Pa significantly correlated with LDH levels (*r* = −0.47, *p* = 0.004; *r* = −0.63; *p* < 0.001; respectively). No significant difference was observed between the two groups for the coagulation markers (protein C, protein S, D-dimer).

**Table 3:** Rheological and biological characteristics according to nocturnal hypoxemia.

	**No nocturnal hypoxaemia (*n* = 36)**	**Nocturnal hypoxoemia (*n* = 7)**	***p***
RBC deformability index at 3 Pa (UI)	0.26 ± 0.05	0.18 ± 0.09	**0.04**
RBC deformability index at 30 Pa (UI)	0.47 ± 0.08	0.32 ± 0.13	**0.03**
Blood viscosity at 22.5 s-1 (cP)	18.1 ± 18	8.5 ± 7.3	**0.039**
Blood viscosity at 45 s-1 (cP)	11.9 ± 10.3	6.3 ± 5.2	**0.049**
Hb (g/L)	91.1 ± 15.8	76.7 ± 20.8	**0.046**
HbF (%)	14.8 ± 8.5	8.2 ± 5.6	ns
Ht (%)	26.1 ± 4.1	22.1 ± 6.4	**0.05**
Neutrophils (G/L)	4.4 ± 2.5	6.6 ± 3.1	**0.047**
Moncytes (G/L)	1 ± 0.4	1.7 ± 1	**0.002**
Reticulocytes (%)	9.2 ± 4	20.1 ± 8	**0.011**
Free bilirubin (μmol/L)	34.1 ± 26	87 ± 44.6	**0.001**
LDH (U/L)	356.1 ± 107.5	551.8 ± 89.5	**0.001**
C protein (%)	78.1 ± 13.8	69.5 ± 17.6	ns
S protein (%)	62.7 ± 15.9	66.7 ± 10.7	ns
D-dimer (μg/L)	2255 ± 1731.9	2988.3 ± 2025.8	ns

*RBC, Red blood cell; Hb, Hemoglobin concentration; HbF, hemoglobin F; HbS, hemoglobin S; Ht, hematocrit; ns, non significant. Bold p values mean Statistically significant*.

#### Effects of OSA

Data are displayed in Table 4. No significant difference was observed between OSA and non-OSA patients for the different biological parameters.

**Table 4:** Rheological and biological characteristics according to OSA.

	**Non OSA (*n* = 14)**	**OSA (*n* = 29)**	***p***
RBC deformability index at 3 Pa (UI)	0.27 ± 0.04	0.23 ± 0.07	ns
RBC deformability index at 30 Pa (UI)	0.47 ± 0.07	0.42 ± 0.12	ns
Blood viscosity at 22.5 s-1 (cP)	18.2 ± 19.1	15.6 ± 16.3	ns
Blood viscosity at 45 s-1 (cP)	10 ± 7.9	11.4 ± 10.7	ns
Hb (g/L)	92.2 ± 14.8	86.7 ± 18.5	ns
HbF (%)	13.3 ± 7.3	13.9 ± 9.1	ns
Ht (%)	26.1 ± 3.9	24 ± 5.5	ns
Neutrophils (G/L)	4.7 ± 2.5	4.9 ± 2.8	ns
Monocytes (G/L)	1.2 ± 0.5	1.1 ± 0.6	ns
Reticulocytes (%)	13.6 ± 7.2	10 ± 5.9	ns
Free bilirubin (μmol/L)	39.2 ± 25.6	43.2 ± 37.8	ns
LDH (U/L)	394.1 ± 121.6	377.9 ± 128.4	ns
C protein (%)	78.3 ± 15.7	76.1 ± 14.2	ns
S protein (%)	62.7 ± 15.8	64.8 ± 20.2	ns
D-dimer (μg/L)	2942.7 ± 2394.8	2082 ± 1267.6	ns

*RBC, Red blod cell; Hb, Hemoglobin concentration; HbF, hemoglobin F; HbS, hemoglobin S; Ht, hematocrit; ns, non significant*.

## Discussion

Our study demonstrated that: (1) nocturnal hypoxemia and OSA were not associated; (2) nocturnal hypoxemia but not OSA was accompanied by a decrease in RBC deformability, enhanced hemolysis and more severe anemia in SCD patients and (3) nocturnal hypoxemia but not OSA was associated with a higher proportion of patients with hemolytic complications.

The hallmark of OSA is recurrent episodes of hypoxemia and arousals throughout the night (Gottlieb and Young, 2009; Dempsey et al., 2010). As a result, OSA has been suspected to trigger vaso-occlusive like events in SCD (Hargrave et al., 2003; Gileles-Hillel et al., 2015; Brennan et al., 2020). However, our study clearly showed that significant nocturnal hypoxemia may occur independently of OSA, which confirm previous findings (Whitesell et al., 2016; Worsham et al., 2017). Respiratory events from our OSA subjects were probably not sufficiently long and deep to generate hypoxemia. It could be explained by the fact that our population study is relatively young (Ware et al., 2000). Moreover, OSA was not accompanied by an increased rate of vaso-occlusive like complications, which is in agreement with the study of Willen et al. (2018). Lung function was found to be not different between OSA and non-OSA patients, as well as between hypoxemic and non-hypoxemic individuals. Indeed, the lower oxyhemoglobin saturation found in some SCD patients in this study seems to be not due to impaired lung diffusion, which is in agreement with a previous study showing no association between pulmonary function and the prevalence of nocturnal oxyhemoglobin desaturation in SCD children (Needleman et al., 1999).

Since HbS may polymerize under deoxygenation, the rheology of RBCs from SCD patients is highly sensitive to the level of blood oxygen. Boisson et al. (2021) recently demonstrated that RBC sickling may occur in some patients at oxygen tensions of 70–80 mmHg, which is in agreement with the previous finding of Nash et al. (1986) who observed RBC sickling at oxygen tension >60 mmHg. Lu et al. (2016) reported a decrease of flow velocity of RBC from SCD patients in a microfluidic device when oxygen tensions approached 70 mmHg, which was consistent with a rise in blood viscosity caused by the formation of sickled RBC. Moreover, repeated sickling—unsickling cycles when RBCs circulate from low to high oxygen tension vascular areas cause permanent damages to the membrane, which impairs the mean ability of RBCs to deform (Padilla et al., 1973). Indeed, one may suspect that significant hypoxemia would permanently change the rheology of RBC in SCD patients. Our results showed that patients with nocturnal hypoxemia had lower mean RBC deformability than patients without nocturnal hypoxemia. In addition, patients with nocturnal hypoxemia had high reticulocytes count, LDH level and bilirubin, which indicate enhanced hemolysis that would be at the origin of the lower Hb concentration in this group. The correlations found between RBC deformability and LDH levels suggest that the most rigid RBCs would be more fragile than the most deformable ones. Connes et al. (2014) have shown that dense and rigid sickle RBCs are prone to greater mechanical fragmentation when exposed to continuous shear stress, as it is the case in the blood circulation, compared to less dense and more deformable RBCs, indicating that a direct link between decreased RBC deformability and increased hemolysis exist in SCD.

Through its effects on inflammation and oxidative stress, hemolysis plays a central role in the pathophysiology of SCD and participates to the development of progressive vasculopathy and organ damages (Kato et al., 2018; Nader et al., 2021). Coagulation markers were not increased in patients with nocturnal hypoxemia but the greater monocytes and neutrophils count suggest that inflammation could be slightly increased in comparison with the non-hypoxemic group. Two main biological-clinical phenotypes would co-exist in SCD (Kato et al., 2018), with patients with the greatest hemolytic rate being prone to develop leg ulcers, priapism, glomerulopathy, pulmonary hypertension and cerebral vasculopathy, and patients with the lowest hemolytic rate having enhanced blood viscosity that would increase the risk for developing vaso-occlusive like complications, such as VOC, ACS, osteonecrosis, and retinopathy. Although this model is debated (Hebbel, 2011) and sometimes difficult to apply in African countries (Dubert et al., 2017), consistent data have been reported in European and US countries that partly support this pathophysiological scheme (Ballas, 1991; Nolan et al., 2005, 2006; Gladwin and Vichinsky, 2008; Gurkan et al., 2010; Maier-Redelsperger et al., 2010; Connes et al., 2013). Our results confirm the link already reported between oxygen hemoglobin saturation, hemolysis, and anemia (Homi et al., 1997; Quinn and Ahmad, 2005; Rotz et al., 2016) and suggest that nocturnal hypoxemia could play a key role in the development of complications from the hemolytic phenotype.

Alternatively, one could also hypothesize that a further decrease in red blood cell deformability could participate in the occurrence or strengthening of hypoxemia, as it has already been suggested in non-SCD individuals (Connes et al., 2004). Any decrease in RBC deformability has been demonstrated to impair blood flow dynamics in the pulmonary circulation of animals (Hakim, 1988) and could favor thrombosis (Gillespie and Doctor, 2021). Pulmonary thrombotic arteriopathy has been reported in SCD patients (Adedeji et al., 2001). The resulting alterations in blood flow dynamics may lead to intrapulmonary shunts (Hambley et al., 2019), a situation previously described in SCD patients (Desai et al., 2019; Hambley et al., 2019), which could cause hypoxemia of unclear etiology.

In conclusion, nocturnal hypoxemia could be responsible for changes in RBC deformability that would fragilize RBCs resulting in enhanced hemolysis that could play a significant role in the development of complications such as leg ulcers, priapism, pulmonary hypertension or glomerulopathy. The identification of nocturnal hypoxemia and its causes, other than OSA, and treatment of SDB could be helpful to decrease morbidity in SCD patients.

## Data Availability Statement

The raw data supporting the conclusions of this article will be made available by the authors, without undue reservation.

## Ethics Statement

The studies involving human participants were reviewed and approved by CPP Lyon OUEST V. Written informed consent to participate in this study was provided by the participants' legal guardian/next of kin.

## Author Contributions

ES and PC designed the study and performed statistical analyses and wrote the first version of the manuscript. SP, GC, AG, RF, YB, and AH recruited the patients and collected clinical data. ES, CR, PJ, CB, VP, and PC performed biological analyses. ES, LP-D, and TP performed sleep-related measurements. PC is the guarantor of this study. All authors read and approved the manuscript before submission.

## Conflict of Interest

The authors declare that the research was conducted in the absence of any commercial or financial relationships that could be construed as a potential conflict of interest.

## Publisher's Note

All claims expressed in this article are solely those of the authors and do not necessarily represent those of their affiliated organizations, or those of the publisher, the editors and the reviewers. Any product that may be evaluated in this article, or claim that may be made by its manufacturer, is not guaranteed or endorsed by the publisher.
